# Supplementary material for: From tests to truth: A misclassification-aware machine learning framework for estimating brucellosis seroprevalence in wild canids
Source: PLoS Negl Trop Dis. 2026 Mar 6;20(3):e0014029. doi: 10.1371/journal.pntd.0014029 (PMC12965539; doi:10.1371/journal.pntd.0014029)
Supplement: S1 Fig — (DOCX) [file pntd.0014029.s002.docx]

**S1 Fig. Risk-of-Bias Traffic-Light Heatmap for All Included Studies**


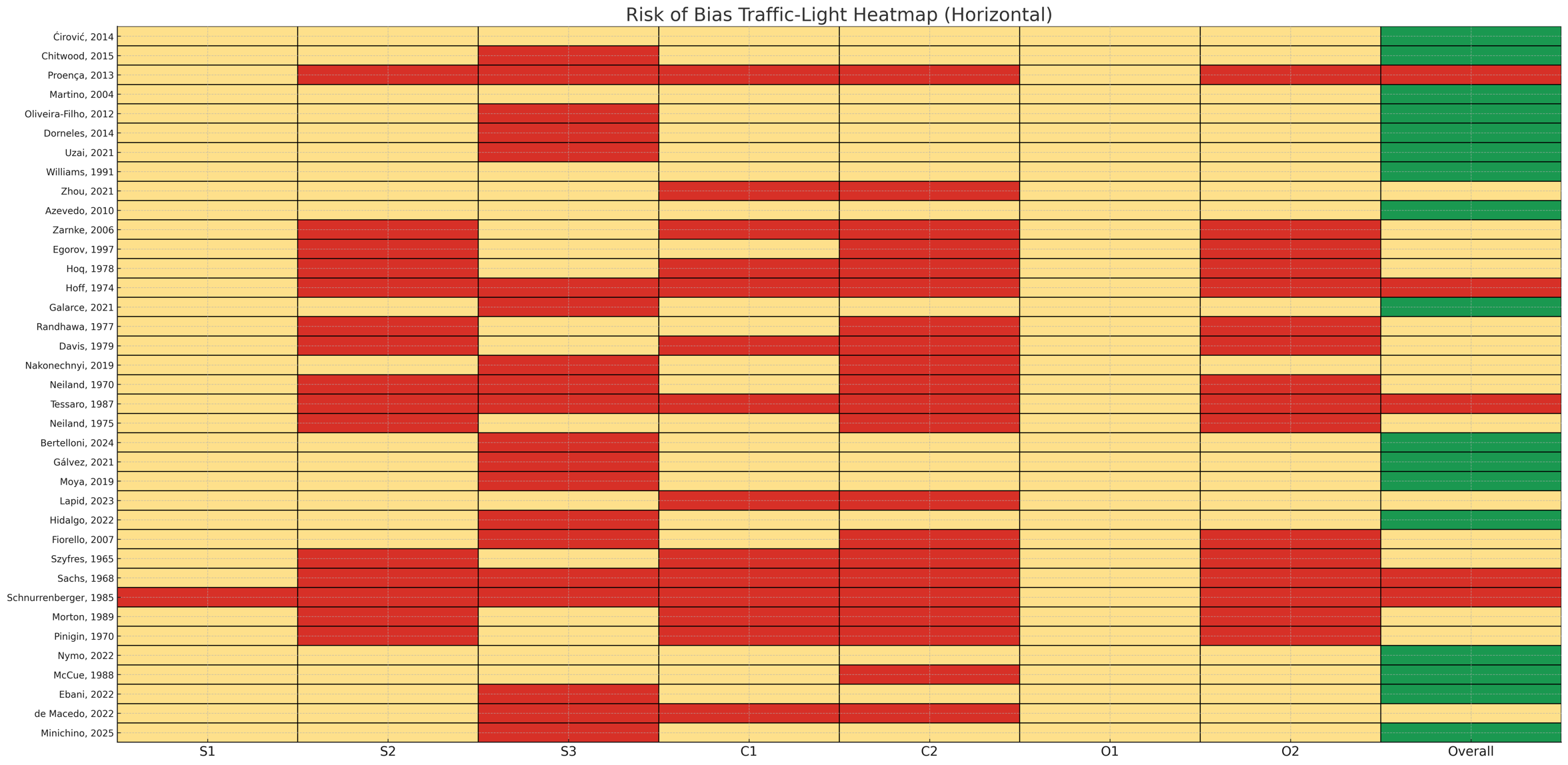


**S1 Fig.** Traffic-light heatmap summarizing the item-level risk-of-bias assessment for all 37 included studies using the adapted seven-item Newcastle–Ottawa Scale (NOS). Each column represents one NOS domain (S1–S3: selection; C1–C2: diagnostic validity; O1–O2: outcome reporting), and each row corresponds to a single study. Cells are color-coded as **green = low risk (criterion met; score = 1), yellow = moderate/unclear risk (partially met or insufficient detail), and red = high risk (criterion not met; score = 0).** The final column (“Overall”) shows the overall risk-of-bias category derived from total QA scores (low: 5–7; moderate: 3–4; high: 0–2). Full numerical scores for each domain are provided in S2 Table.

.
