# Supplementary material for: From tests to truth: A misclassification-aware machine learning framework for estimating brucellosis seroprevalence in wild canids
Source: PLoS Negl Trop Dis. 2026 Mar 6;20(3):e0014029. doi: 10.1371/journal.pntd.0014029 (PMC12965539; doi:10.1371/journal.pntd.0014029)
Supplement: S2 File — (DOCX) [file pntd.0014029.s003.docx]

### ****S2 File. Full Database Search Strings****

This supplementary file provides the complete search strings for each database used in the scoping review. Search terms were organized around four conceptual domains: (1) disease-related terminology, (2) host taxa (wild canids), (3) epidemiological descriptors, and (4) diagnostic methods. Controlled vocabulary (e.g., MeSH/DeCS terms) was combined with free-text terms to maximize sensitivity. **No language restrictions were applied during the search; all records retrieved through the databases were screened regardless of publication language.** The final included studies were published in English, Persian, Spanish, Portuguese, or Russian, reflecting the composition of the evidence retrieved rather than imposed language limits.

**PubMed**
**Search date:** July 12, 2025
**Search string:**
("Brucellosis"[Mesh] OR "Brucella abortus"[Mesh] OR "Brucella suis"[Mesh] OR "Brucella canis"[Mesh] OR brucellosis OR Brucella)
AND
("Canidae"[Mesh] OR canids OR wild canids OR foxes OR wolves OR jackals OR coyotes OR wild dogs)
AND
(prevalence OR seroprevalence OR epidemiology OR serology OR ELISA OR PCR OR culture)

**Scopus**
**Search date:** July 15, 2025
**Search string:**
TITLE-ABS-KEY (brucellosis OR "Brucella abortus" OR "Brucella suis" OR "Brucella canis")
AND
TITLE-ABS-KEY (canids OR "wild canids" OR foxes OR wolves OR jackals OR coyotes OR "wild dogs")
AND
TITLE-ABS-KEY (prevalence OR seroprevalence OR serology OR PCR OR culture OR diagnostics)

**Web of Science – Core Collection**
**Search date:** July 17, 2025
**Search string:**
TS=(brucellosis OR "Brucella abortus" OR "Brucella suis" OR "Brucella canis")
AND
TS=(canids OR "wild canids" OR foxes OR wolves OR jackals OR coyotes OR "wild dogs")
AND
TS=(prevalence OR seroprevalence OR serology OR PCR OR culture OR diagnostics)

**SciELO**
**Search date:** July 20, 2025
**Search string:**
(brucelose OR brucellosis OR "Brucella abortus" OR "Brucella suis")
AND
(cães selvagens OR canídeos OR foxes OR lobos OR chacais OR coiotes)
AND
(prevalência OR prevalencia OR prevalence OR serologia OR PCR OR cultura)

**Google Scholar**
**Search date:** July 22, 2025
**Search string:**
brucellosis "wild canids" OR foxes OR wolves OR coyotes OR jackals OR wild dogs prevalence serology PCR culture

**Screening approach:** The first 200 search results were manually screened by title and abstract. This threshold was determined through pilot testing, which showed diminishing relevance beyond this point.
