# Supplementary material for: From tests to truth: A misclassification-aware machine learning framework for estimating brucellosis seroprevalence in wild canids
Source: PLoS Negl Trop Dis. 2026 Mar 6;20(3):e0014029. doi: 10.1371/journal.pntd.0014029 (PMC12965539; doi:10.1371/journal.pntd.0014029)
Supplement: S1 Table — (DOCX) [file pntd.0014029.s004.docx]

**S1 Table. Full-text Screening Exclusion Log (n = 53)**
Each excluded record (EX-01 to EX-53) is listed with the primary reason for exclusion. In cases where multiple issues were identified, all applicable reasons are noted.

| **Record ID** | **Reason(s) for exclusion** |
| --- | --- |
| EX-01 | No original prevalence data |
| EX-02 | Domestic/zoo animals; unclear diagnostic method |
| EX-03 | Unrepresentative sample size |
| EX-04 | Incomplete methodological reporting |
| EX-05 | No original prevalence data |
| EX-06 | Unclear diagnostic methods |
| EX-07 | Domestic/zoo animals |
| EX-08 | No original prevalence data |
| EX-09 | Sample size too small |
| EX-10 | Unclear diagnostic methods; incomplete reporting |
| EX-11 | No original prevalence data |
| EX-12 | Domestic/zoo animals |
| EX-13 | No original prevalence data |
| EX-14 | No original prevalence data; unclear methods |
| EX-15 | Domestic/zoo animals |
| EX-16 | Sample size too small |
| EX-17 | Incomplete methodological reporting |
| EX-18 | No original prevalence data |
| EX-19 | Unclear diagnostic methods |
| EX-20 | No original prevalence data |
| EX-21 | Domestic/zoo animals; sample too small |
| EX-22 | No original prevalence data |
| EX-23 | Unclear diagnostic methods |
| EX-24 | Sample size too small or unrepresentative |
| EX-25 | Domestic/zoo animals |
| EX-26 | No original prevalence data; incomplete reporting |
| EX-27 | Domestic/zoo animals |
| EX-28 | Unclear diagnostic methods |
| EX-29 | No original prevalence data |
| EX-30 | Sample size too small |
| EX-31 | Incomplete methodological reporting |
| EX-32 | Domestic/zoo animals |
| EX-33 | No original prevalence data |
| EX-34 | Unclear diagnostic methods |
| EX-35 | Sample size too small |
| EX-36 | Domestic/zoo animals |
| EX-37 | No original prevalence data |
| EX-38 | Incomplete methodological reporting |
| EX-39 | Unclear diagnostic methods |
| EX-40 | No original prevalence data; sample too small |
| EX-41 | Domestic/zoo animals |
| EX-42 | Incomplete methodological reporting |
| EX-43 | Unrepresentative sample size |
| EX-44 | No original prevalence data |
| EX-45 | Unclear diagnostic methods |
| EX-46 | Domestic/zoo animals; unclear methods |
| EX-47 | Sample size too small |
| EX-48 | No original prevalence data |
| EX-49 | Incomplete methodological reporting |
| EX-50 | Domestic/zoo animals |
| EX-51 | Unclear diagnostic methods |
| EX-52 | No original prevalence data |
| EX-53 | Sample size too small; incomplete reporting |
