# Supplementary material for: From tests to truth: A misclassification-aware machine learning framework for estimating brucellosis seroprevalence in wild canids
Source: PLoS Negl Trop Dis. 2026 Mar 6;20(3):e0014029. doi: 10.1371/journal.pntd.0014029 (PMC12965539; doi:10.1371/journal.pntd.0014029)
Supplement: S2 Table — (DOCX) [file pntd.0014029.s005.docx]

**S2 Table. Risk of Bias Assessment Using the Adapted Newcastle–Ottawa Scale (NOS)**

| **Study** | **S1- Sampling Frame Representativeness (0/1)** | **S2- Clear Inclusion / Exclusion Criteria (0/1)** | **S3- Adequate Sample Size Reported (0/1)** | **C1- Validated Diagnostic Test Used (0/1)** | **C2- Diagnostic Protocol & Cut-offs Reported (0/1)** | **O1- Clear Numerator / Denominator (0/1)** | **O2- Outcome Reporting Reproducible (0/1)** | **QA Score (0–7)** | **Risk of Bias (Low / Moderate / High)** |
| --- | --- | --- | --- | --- | --- | --- | --- | --- | --- |
| **Study** | 1 | 1 | 1 | 1 | 1 | 1 | 1 | 7 | **Low** |
| Ćirović et al. [1] | 1 | 1 | 0 | 1 | 1 | 1 | 1 | 6 | **Low** |
| Chitwood et al. [2] | 1 | 0 | 0 | 0 | 0 | 1 | 0 | 2 | **High** |
| Proença et al. [3] | 1 | 1 | 1 | 1 | 1 | 1 | 1 | 7 | **Low** |
| Martino et al. [4] | 1 | 1 | 0 | 1 | 1 | 1 | 1 | 6 | **Low** |
| Oliveira-Filho et al. [5] | 1 | 1 | 0 | 1 | 1 | 1 | 1 | 6 | **Low** |
| Dorneles et al. [6] | 1 | 1 | 0 | 1 | 1 | 1 | 1 | 6 | **Low** |
| Uzai et al. [7] | 1 | 1 | 1 | 1 | 1 | 1 | 1 | 7 | **Low** |
| Williams et al. [8] | 1 | 1 | 1 | 0 | 0 | 1 | 1 | 5 | **Moderate** |
| Zhou et al. [9] | 1 | 1 | 1 | 1 | 1 | 1 | 1 | 7 | **Low** |
| Azevedo et al. [10] | 1 | 0 | 1 | 0 | 0 | 1 | 0 | 3 | **Moderate** |
| Zarnke et al. [11] | 1 | 0 | 1 | 1 | 0 | 1 | 0 | 4 | **Moderate** |
| Egorov et al. [12] | 1 | 0 | 1 | 0 | 0 | 1 | 0 | 3 | **Moderate** |
| Hoq [13] | 1 | 0 | 0 | 0 | 0 | 1 | 0 | 2 | **High** |
| Hoff et al. [14] | 1 | 1 | 0 | 1 | 1 | 1 | 1 | 6 | **Low** |
| Galarce et al. [15] | 1 | 0 | 1 | 1 | 0 | 1 | 0 | 4 | **Moderate** |
| Randhawa et al. [16] | 1 | 0 | 1 | 0 | 0 | 1 | 0 | 3 | **Moderate** |
| Davis et al. [17] | 1 | 1 | 0 | 1 | 0 | 1 | 1 | 5 | **Moderate** |
| Nakonechnyi et al. [18] | 1 | 0 | 0 | 1 | 0 | 1 | 0 | 3 | **Moderate** |
| Neiland [19] | 1 | 0 | 0 | 0 | 0 | 1 | 0 | 2 | **High** |
| Tessaro [20] | 1 | 0 | 1 | 1 | 0 | 1 | 0 | 4 | **Moderate** |
| Neiland [21] | 1 | 1 | 0 | 1 | 1 | 1 | 1 | 6 | **Low** |
| Bertelloni et al. [22] | 1 | 1 | 0 | 1 | 1 | 1 | 1 | 6 | **Low** |
| Fuente González [23] | 1 | 1 | 0 | 1 | 1 | 1 | 1 | 6 | **Low** |
| Moya et al. [24] | 1 | 1 | 1 | 0 | 0 | 1 | 1 | 5 | **Moderate** |
| Lapid et al. [25] | 1 | 1 | 0 | 1 | 1 | 1 | 1 | 6 | **Low** |
| Hidalgo-Hermoso et al. [26] | 1 | 1 | 0 | 1 | 0 | 1 | 0 | 4 | **Moderate** |
| Fiorello et al. [27] | 1 | 0 | 1 | 0 | 0 | 1 | 0 | 3 | **Moderate** |
| Szyfres & González Tomé [28] | 1 | 0 | 0 | 0 | 0 | 1 | 0 | 2 | **High** |
| Sachs et al. [29] | 0 | 0 | 0 | 0 | 0 | 1 | 0 | 1 | **High** |
| Schnurrenberger et al. [30] | 1 | 0 | 1 | 0 | 0 | 1 | 0 | 3 | **Moderate** |
| Morton [31] | 1 | 0 | 1 | 0 | 0 | 1 | 0 | 3 | **Moderate** |
| Pinigin et al. [32] | 1 | 1 | 1 | 1 | 1 | 1 | 1 | 7 | **Low** |
| Nymo et al. [33] | 1 | 1 | 1 | 1 | 0 | 1 | 1 | 6 | **Low** |
| McCue & O’Farrell [34] | 1 | 1 | 0 | 1 | 1 | 1 | 1 | 6 | **Low** |
| Ebani et al. [35] | 1 | 1 | 0 | 0 | 0 | 1 | 1 | 4 | **Moderate** |
| de Macedo et al. [36] | 1 | 1 | 0 | 1 | 1 | 1 | 1 | 6 | **Low** |
| Minichino et al. [37] | 1 | 1 | 0 | 1 | 1 | 1 | 1 | 6 | **Low** |

**S2 Table presents the item-level quality appraisal for all included studies, evaluated using an adapted Newcastle–Ottawa Scale (NOS) designed for prevalence studies of wildlife infections.** The tool comprises seven binary criteria covering three domains: selection (S1–S3), comparability/diagnostic quality (C1–C2), and outcome reporting (O1–O2). Each criterion is scored as **1 = criterion met** or **0 = criterion not met.** The total Quality Appraisal (QA) Score is calculated as the sum of all item scores, yielding a range from **0 to 7.**

**Domain definitions:**

- **S1 – Sampling frame representativeness:** The sampled animals reasonably reflect the underlying wild population.
- **S2 – Clear inclusion/exclusion criteria:** Eligibility criteria explicitly described.
- **S3 – Adequate sample size reported:** Sample size (N) clearly stated and sufficient to interpret prevalence estimates.
- **C1 – Validated diagnostic test used:** Use of recognized assays such as ELISA, CFT, PCR, or culture.
- **C2 – Diagnostic protocol and cut-offs reported:** Diagnostic procedures, antigens, or interpretive thresholds adequately described.
- **O1 – Clear numerator/denominator reporting:** Both number tested (N) and number positive (n) clearly stated.
- **O2 – Outcome reporting reproducible:** Data reported with enough detail for independent verification or re-analysis.

**Interpretation of QA Scores:**

- **Low risk of bias:** 5–7
- **Moderate risk of bias:** 3–4
- **High risk of bias:** 0–2

Quality assessment did not influence study inclusion. Instead, these evaluations informed interpretation of the evidence base and supported sensitivity analyses. This table provides full transparency regarding methodological strengths and limitations across studies contributing to the review.

**References**

1. Ćirović D, Chochlakis D, Tomanović S, Sukara R, Penezić A, Tselentis Y, et al. Presence of Leishmania and Brucella species in the golden jackal (Canis aureus) in Serbia. Biomed Res Int. 2014;2014:728516.

2. Chitwood MC, Swingen MB, Lashley MA, Flowers JR, Palamar MB, Apperson CS, et al. Parasitology and serology of free-ranging coyotes (Canis latrans) in North Carolina, USA. J Wildl Dis. 2015;51(3):664–669.

3. Proença LM, Silva JC, Galera PD, Lion MB, Marinho-Filho JS, Ragozo AMA, et al. Serologic survey of infectious diseases in populations of maned wolf (Chrysocyon brachyurus) and crab-eating fox (Cerdocyon thous) from Águas Emendadas Ecological Station, Brazil. J Zoo Wildl Med. 2013;44:152–155.

4. Martino PE, Montenegro JL, Preziosi JA, Venturini C, Bacigalupe D, Stanchi NO, et al. Serological survey of selected pathogens of free-ranging foxes in southern Argentina, 1998–2001. Rev Sci Tech. 2004;23:801–806.

5. Oliveira-Filho EF, Júnior JWP, Souza MM, Santana VL, Silva JC, Mota RA, et al. Serologic survey of brucellosis in captive neotropical wild carnivores in northeast Brazil. J Zoo Wildl Med. 2012;43(2):384–387.

6. Dorneles EMS, Pellegrin AO, Péres IAHFS, Mathias LA, Mourão G, Bianchi R de C, et al. Serology for brucellosis in free-ranging crab-eating foxes (Cerdocyon thous) and brown-nosed coatis (Nasua nasua) from Brazilian Pantanal. Cienc Rural. 2014;44(12):2193–2196.

7. Uzai GJS, Monteiro CP, Soares R, Silva MA, Oliveira AR, Santos DO, et al. Morphological and molecular diagnosis of diseases of free-ranging crab-eating foxes (Cerdocyon thous). Arq Bras Med Vet Zootec. 2021;73(3):583–588.

8. Williams JD, Heck FC, Davis DS, Adams LG. Comparison of results from five serologic methods used for detecting Brucella abortus antibody activity in coyote sera. Vet Immunol Immunopathol. 1991;29(1–2):79–87.

9. Zhou Y, Meng Y, Ren Y, Liu Z, Li Z. A retrospective survey of the abortion outbreak event caused by brucellosis at a blue fox breeding farm in Heilongjiang Province, China. Front Vet Sci. 2021;8:666254.

10. Azevedo SS de, Silva MLCR, Batista C de SA, Gomes AA de B, Vasconcellos SA, Alves CJ. Detection of anti-Brucella abortus, anti-Brucella canis, and anti-Leptospira spp. antibodies in hoary foxes (Pseudalopex vetulus) from semi-arid of Paraiba State, Northeastern region of Brazil. Cienc Rural. 2010;40(1):190–192.

11. Zarnke RL, Ver Hoef JM, DeLong RA. Geographic pattern of serum antibody prevalence for Brucella spp. in caribou, grizzly bears, and wolves from Alaska, 1975–1998. J Wildl Dis. 2006;42(3):570–577.

12. Egorov EA, Kalinovski AI, Maramovich AS, Cherniavski VF. Problems of epidemiologic surveillance of brucellosis in reindeer breeding. Epidemiol Infect Dis. 1997;2:18–21.

13. Hoq MA. A serologic survey of Brucella agglutinins in wildlife and sheep. Calif Vet. 1978;32(3):15–17.

14. Hoff GL, Bigler WJ, Trainer DO, Debbie JG, Brown GM, Winkler WG, et al. Survey of selected carnivore and opossum serums for agglutinins to Brucella canis. J Am Vet Med Assoc. 1974;165(9):830–831.

15. Galarce N, de la Fuente S, Escobar B, Dettleff P, Abalos P, Hormazábal JC, et al. Survey of zoonotic bacterial pathogens in native foxes in Central Chile: First record of Brucella canis exposure. Animals (Basel). 2021;11(7):1980.

16. Randhawa AS, Kelly VP, Baker EF. Agglutinins to Coxiella burnetii and Brucella spp., with particular reference to Brucella canis, in wild animals of southern Texas. J Am Vet Med Assoc. 1977;171(9):939–942.

17. Davis DS, Boeer WJ, Mims JP, Heck FC, Adams LG. Brucella abortus in coyotes. I. A serologic and bacteriologic survey in eastern Texas. J Wildl Dis. 1979;15(3):367–372.

18. Nakonechnyi IV, Perots’ka LV, Pyvovarova IV, Chornyi VA. Ecological and epizootic roles of the golden jackal (Canis aureus) in the northwest of the Black Sea coast. Sci Messenger LNU Vet Med Biotechnol Vet Sci. 2019;21(94):37–43.

19. Neiland KA. Rangiferine brucellosis in Alaskan canids. J Wildl Dis. 1970;6(3):136–139.

20. Tessaro SV. A descriptive and epizootiologic study of brucellosis and tuberculosis in bison in northern Canada. PhD dissertation. Saskatoon: University of Saskatchewan; 1987.

21. Neiland KA. Further observations on rangiferine brucellosis in Alaskan carnivores. J Wildl Dis. 1975;11(1):45–53.

22. Bertelloni F, Cagnoli G, Ebani VV. Survey on the occurrence of zoonotic bacterial pathogens in the feces of wolves (Canis lupus italicus) collected in a protected area in central Italy. Microorganisms. 2024;12:2367.

23. Fuente González SI de la. Detección de la infección por Brucella abortus y Brucella canis en cánidos no domésticos de centro de rehabilitación y exhibición de la zona central de Chile. Bachelor’s thesis. Santiago: Universidad de Chile; 2021.

24. Moya S, Oettinger S, Borie C, Flores R, Abalos P, Briceño C. Serologic survey of Brucella canis and Leptospira spp. in free-ranging wild and domestic canids from Tierra del Fuego, Chile. J Wildl Dis. 2019;55(3):713–716.

25. Lapid R, Motro Y, Craddock H, Khalfin B, King R, Bar-Gal GK, et al. Fecal microbiota of the synanthropic golden jackal (Canis aureus). Anim Microbiome. 2023;5:37.

26. Hidalgo-Hermoso E, Cabello J, Verasay J, Moreira-Arce D, Hidalgo M, Abalos P, et al. Serosurvey for selected parasitic and bacterial pathogens in Darwin’s fox (Lycalopex fulvipes): not only dog diseases are a threat. J Wildl Dis. 2022;58(1):76–85.

27. Fiorello CV, Noss AJ, Deem SL, Maffei L, Dubovi EJ. Serosurvey of small carnivores in the Bolivian Chaco. J Wildl Dis. 2007;43(3):551–557.

28. Szyfres B, González Tomé J. Natural Brucella infection in Argentine wild foxes. Bull World Health Organ. 1966;34(6):919–923.

29. Sachs R, Staak C, Groocock CM. Serological investigation of brucellosis in game animals in Tanzania. Report. Dar es Salaam: Veterinary Research Institute; 1968.

30. Schnurrenberger PR, Brown RR, Hill EP, Scanlan CM, Altiere JA, Wykoff JT. Brucella abortus in wildlife on selected cattle farms in Alabama. J Wildl Dis. 1985;21(1):132–136.

31. Morton JK. Brucella suis Type 4 in foxes and their role as reservoirs and vectors among reindeer. PhD dissertation. Fairbanks (AK): University of Alaska Fairbanks; 1989.

32. Pinigin AF, Zabrodin VA, Nikulina VI. Brucellosis in arctic foxes (Alopex lagopus). Report. Moscow: Academy of Sciences of the USSR; 1970.

33. Nymo IH, Fuglei E, Mørk T, Breines EM, Holmgren KE, Davidson RK, et al. Why are Svalbard Arctic foxes Brucella spp. seronegative? Polar Res. 2022;41:7867.

34. McCue PM, O’Farrell TP. Serological survey for selected diseases in the endangered San Joaquin kit fox (Vulpes macrotis mutica). J Wildl Dis. 1988;24(2):274–281.

35. Ebani VV, Trebino C, Guardone L, Bertelloni F, Cagnoli G, Nardoni S, et al. Occurrence of bacterial and protozoan pathogens in red foxes (Vulpes vulpes) in central Italy. Animals (Basel). 2022;12(18):2891.

36. de Macedo GC, Herrera HM, de Oliveira Porfírio GE, Santos FM, de Assis WO, de Andrade GB, et al. Brucellosis in the Brazilian Pantanal wetland: threat to animal production and wildlife conservation. Braz J Microbiol. 2022;53:2287–2297.

37. Minichino A, Ciuca L, Dipineto L, Rinaldi L, Montagnaro S, Borrelli L, et al. Exposure to selected pathogens in wild mammals from a rescue and rehabilitation center in southern Italy. One Health. 2025;20:101049.
