# Supplementary material for: From tests to truth: A misclassification-aware machine learning framework for estimating brucellosis seroprevalence in wild canids
Source: PLoS Negl Trop Dis. 2026 Mar 6;20(3):e0014029. doi: 10.1371/journal.pntd.0014029 (PMC12965539; doi:10.1371/journal.pntd.0014029)
Supplement: S3 Table — (DOCX) [file pntd.0014029.s006.docx]

**S3 Table.** Summary of included studies in the global meta-analysis of Brucella exposure in wild canids.

| Study | k_pop | Total Positive Cases | Total Tested | Pooled Prevalence (%) | 95% CI – Lower Bound | 95% CI – Upper Bound | Study Weight (N) | Weight Share (%) | I² Within-Study (%) |
| --- | --- | --- | --- | --- | --- | --- | --- | --- | --- |
| Zarnke et al. [1] | 2.00 | 28.00 | 997.00 | 0.03 | 0.02 | 0.04 | 997.00 | 0.20 | 0.00 |
| Szyfres & González Tomé [2] | 1.00 | 173.00 | 728.00 | 0.24 | 0.21 | 0.27 | 728.00 | 0.14 | 0.00 |
| Zhou et al. [3] | 1.00 | 493.00 | 726.00 | 0.68 | 0.64 | 0.71 | 726.00 | 0.14 | 0.00 |
| Pinigin et al. [4] | 2.00 | 7.00 | 428.00 | 0.02 | 0.01 | 0.03 | 428.00 | 0.08 | 0.00 |
| Nymo et al. [5] | 1.00 | 0.00 | 406.00 | 0.00 | 0.00 | 0.01 | 406.00 | 0.08 | 0.00 |
| Randhawa et al. [6] | 1.00 | 16.00 | 198.00 | 0.08 | 0.05 | 0.13 | 198.00 | 0.04 | 0.00 |
| Hoff et al. [7] | 4.00 | 3.00 | 190.00 | 0.02 | 0.00 | 0.05 | 190.00 | 0.04 | 0.00 |
| Sachs et al. [8] | 2.00 | 17.00 | 180.00 | 0.09 | 0.06 | 0.15 | 180.00 | 0.04 | 90.69 |
| Hoq [9] | 1.00 | 9.00 | 148.00 | 0.06 | 0.03 | 0.11 | 148.00 | 0.03 | 0.00 |
| McCue & O’Farrell [10] | 2.00 | 9.00 | 121.00 | 0.07 | 0.03 | 0.14 | 121.00 | 0.02 | 0.00 |
| Williams et al. [11] | 1.00 | 29.00 | 94.00 | 0.31 | 0.22 | 0.41 | 94.00 | 0.02 | 0.00 |
| Martino et al. [12] | 2.00 | 15.00 | 84.00 | 0.18 | 0.10 | 0.28 | 84.00 | 0.02 | 63.82 |
| Morton [13] | 1.00 | 12.00 | 64.00 | 0.19 | 0.10 | 0.30 | 64.00 | 0.01 | 0.00 |
| Azevedo et al. [14] | 1.00 | 16.00 | 60.00 | 0.27 | 0.16 | 0.40 | 60.00 | 0.01 | 0.00 |
| Chitwood et al. [15] | 2.00 | 0.00 | 58.00 | 0.00 | 0.00 | 0.06 | 58.00 | 0.01 | 0.00 |
| Lapid et al. [16] | 1.00 | 0.00 | 56.00 | 0.00 | 0.00 | 0.06 | 56.00 | 0.01 | 0.00 |
| Galarce et al. [17] | 4.00 | 5.00 | 53.00 | 0.09 | 0.03 | 0.21 | 53.00 | 0.01 | 0.00 |
| Davis et al. [18] | 1.00 | 9.00 | 51.00 | 0.18 | 0.08 | 0.31 | 51.00 | 0.01 | 0.00 |
| Tessaro [19] | 2.00 | 5.00 | 50.00 | 0.10 | 0.03 | 0.22 | 50.00 | 0.01 | 77.61 |
| Schnurrenberger et al. [20] | 2.00 | 1.00 | 49.00 | 0.02 | 0.00 | 0.11 | 49.00 | 0.01 | 0.00 |
| Neiland [21] | 3.00 | 16.00 | 46.00 | 0.35 | 0.21 | 0.50 | 46.00 | 0.01 | 0.00 |
| Hidalgo-Hermoso et al. [22] | 1.00 | 0.00 | 46.00 | 0.00 | 0.00 | 0.08 | 46.00 | 0.01 | 0.00 |
| Fuente González [23] | 1.00 | 5.00 | 46.00 | 0.11 | 0.04 | 0.24 | 46.00 | 0.01 | 0.00 |
| Oliveira-Filho et al. [24] | 1.00 | 8.00 | 42.00 | 0.19 | 0.09 | 0.34 | 42.00 | 0.01 | 0.00 |
| de Macedo et al. [25] | 1.00 | 5.00 | 38.00 | 0.13 | 0.04 | 0.28 | 38.00 | 0.01 | 0.00 |
| Dorneles et al. [26] | 1.00 | 5.00 | 38.00 | 0.13 | 0.04 | 0.28 | 38.00 | 0.01 | 0.00 |
| Moya et al. [27] | 2.00 | 0.00 | 27.00 | 0.00 | 0.00 | 0.13 | 27.00 | 0.01 | 0.00 |
| Minichino et al. [28] | 2.00 | 2.00 | 22.00 | 0.09 | 0.01 | 0.29 | 22.00 | 0.00 | 0.00 |
| Fiorello et al. [29] | 2.00 | 0.00 | 14.00 | 0.00 | 0.00 | 0.23 | 14.00 | 0.00 | 0.00 |
| Proença et al. [30] | 2.00 | 0.00 | 10.00 | 0.00 | 0.00 | 0.31 | 10.00 | 0.00 | 0.00 |

Study-level pooled seroprevalence estimates of Brucella exposure in wild canids (n = 30 studies). Columns indicate the number of sampled populations (k_pop), positive cases, total tested individuals, misclassification-adjusted pooled prevalence (%), 95% confidence intervals (CI), and study weight (N). Estimates demonstrate considerable geographic and taxonomic heterogeneity across the global dataset.

**References**

1. Zarnke RL, Ver Hoef JM, DeLong RA. Geographic pattern of serum antibody prevalence for *Brucella* spp. in caribou, grizzly bears, and wolves from Alaska, 1975–1998. J Wildl Dis. 2006;42(3):570–577.
2. Szyfres B, González Tomé J. Natural *Brucella* infection in Argentine wild foxes. Bull World Health Organ. 1966;34(6):919–923.
3. Zhou Y, Meng Y, Ren Y, Liu Z, Li Z. A retrospective survey of the abortion outbreak event caused by brucellosis at a blue fox breeding farm in Heilongjiang Province, China. Front Vet Sci. 2021;8:666254.
4. Pinigin AF, Zabrodin VA, Nikulina VI. Brucellosis in arctic foxes (*Alopex lagopus*) [report]. Moscow: Academy of Sciences of the USSR; 1970.
5. Nymo IH, Fuglei E, Mørk T, Breines EM, Holmgren KE, Davidson RK, et al. Why are Svalbard Arctic foxes *Brucella* spp. seronegative? Polar Res. 2022;41:7867.
6. Randhawa AS, Kelly VP, Baker EF. Agglutinins to *Coxiella burnetii* and *Brucella* spp., with particular reference to *Brucella canis*, in wild animals of southern Texas. J Am Vet Med Assoc. 1977;171(9):939–942.
7. Hoff GL, Bigler WJ, Trainer DO, Debbie JG, Brown GM, Winkler WG, et al. Survey of selected carnivore and opossum serums for agglutinins to *Brucella canis*. J Am Vet Med Assoc. 1974;165(9):830–831.
8. Sachs R, Staak C, Groocock CM. Serological investigation of brucellosis in game animals in Tanzania [report]. Dar es Salaam: Veterinary Research Institute; 1968.
9. Hoq MA. A serologic survey of *Brucella* agglutinins in wildlife and sheep. Calif Vet. 1978;32(3):15–17.
10. McCue PM, O’Farrell TP. Serological survey for selected diseases in the endangered San Joaquin kit fox (*Vulpes macrotis mutica*). J Wildl Dis. 1988;24(2):274–281.
11. Williams JD, Heck FC, Davis DS, Adams LG. Comparison of results from five serologic methods used for detecting *Brucella abortus* antibody activity in coyote sera. Vet Immunol Immunopathol. 1991;29(1–2):79–87.
12. Martino PE, Montenegro JL, Preziosi JA, Venturini C, Bacigalupe D, Stanchi NO, et al. Serological survey of selected pathogens of free-ranging foxes in southern Argentina, 1998–2001. Rev Sci Tech. 2004;23:801–806.
13. Morton JK. *Brucella suis* Type 4 in foxes and their role as reservoirs and vectors among reindeer [PhD dissertation]. Fairbanks (AK): University of Alaska Fairbanks; 1989.
14. Azevedo SS de, Silva MLCR, Batista CSA, Gomes AAB, Vasconcellos SA, Alves CJ. Detection of anti-*Brucella abortus*, anti-*Brucella canis*, and anti-*Leptospira* spp. antibodies in hoary foxes (*Pseudalopex vetulus*) from semi-arid of Paraiba State, Northeastern region of Brazil. Cienc Rural. 2010;40(1):190–192.
15. Chitwood MC, Swingen MB, Lashley MA, Flowers JR, Palamar MB, Apperson CS, et al. Parasitology and serology of free-ranging coyotes (*Canis latrans*) in North Carolina, USA. J Wildl Dis. 2015;51(3):664–669.
16. Lapid R, Motro Y, Craddock H, Khalfin B, King R, Bar-Gal GK, et al. Fecal microbiota of the synanthropic golden jackal (*Canis aureus*). Anim Microbiome. 2023;5:37.
17. Galarce N, de la Fuente S, Escobar B, Dettleff P, Abalos P, Hormazábal JC, et al. Survey of zoonotic bacterial pathogens in native foxes in Central Chile: First record of *Brucella canis* exposure. Animals (Basel). 2021;11(7):1980.
18. Davis DS, Boeer WJ, Mims JP, Heck FC, Adams LG. *Brucella abortus* in coyotes. I. A serologic and bacteriologic survey in eastern Texas. J Wildl Dis. 1979;15(3):367–372.
19. Tessaro SV. A descriptive and epizootiologic study of brucellosis and tuberculosis in bison in northern Canada [PhD dissertation]. Saskatoon: University of Saskatchewan; 1987.
20. Schnurrenberger PR, Brown RR, Hill EP, Scanlan CM, Altiere JA, Wykoff JT. *Brucella abortus* in wildlife on selected cattle farms in Alabama. J Wildl Dis. 1985;21(1):132–136.
21. Neiland KA. Rangiferine brucellosis in Alaskan canids. J Wildl Dis. 1970;6(3):136–139.
22. Hidalgo-Hermoso E, Cabello J, Verasay J, Moreira-Arce D, Hidalgo M, Abalos P, et al. Serosurvey for selected parasitic and bacterial pathogens in Darwin’s fox (*Lycalopex fulvipes*): not only dog diseases are a threat. J Wildl Dis. 2022;58(1):76–85.
23. Fuente González SI de la. Detección de la infección por *Brucella abortus* y *Brucella canis* en cánidos no domésticos de centro de rehabilitación y exhibición de la zona central de Chile [bachelor’s thesis]. Santiago: Universidad de Chile; 2021.
24. Oliveira-Filho EF, Júnior JWP, Souza MM, Santana VL, Silva JC, Mota RA, et al. Serologic survey of brucellosis in captive neotropical wild carnivores in northeast Brazil. J Zoo Wildl Med. 2012;43(2):384–387.
25. de Macedo GC, Herrera HM, de Oliveira Porfírio GE, Santos FM, de Assis WO, de Andrade GB, et al. Brucellosis in the Brazilian Pantanal wetland: threat to animal production and wildlife conservation. Braz J Microbiol. 2022;53:2287–2297.
26. Dorneles EMS, Pellegrin AO, Péres IAHFS, Mathias LA, Mourão G, Bianchi RC, et al. Serology for brucellosis in free-ranging crab-eating foxes (*Cerdocyon thous*) and brown-nosed coatis (*Nasua nasua*) from Brazilian Pantanal. Cienc Rural. 2014;44(12):2193–2196.
27. Moya S, Oettinger S, Borie C, Flores R, Abalos P, Briceño C. Serologic survey of *Brucella canis* and *Leptospira* spp. in free-ranging wild and domestic canids from Tierra del Fuego, Chile. J Wildl Dis. 2019;55(3):713–716.
28. Minichino A, Ciuca L, Dipineto L, Rinaldi L, Montagnaro S, Borrelli L, et al. Exposure to selected pathogens in wild mammals from a rescue and rehabilitation center in southern Italy. One Health. 2025;20:101049.
29. Fiorello CV, Noss AJ, Deem SL, Maffei L, Dubovi EJ. Serosurvey of small carnivores in the Bolivian Chaco. J Wildl Dis. 2007;43(3):551–557.
30. Proença LM, Silva JC, Galera PD, Lion MB, Marinho-Filho JS, Ragozo AMA, et al. Serologic survey of infectious diseases in populations of maned wolf (*Chrysocyon brachyurus*) and crab-eating fox (*Cerdocyon thous*) from Águas Emendadas Ecological Station, Brazil. J Zoo Wildl Med. 2013;44:152–155.
