# Supplementary material for: From tests to truth: A misclassification-aware machine learning framework for estimating brucellosis seroprevalence in wild canids
Source: PLoS Negl Trop Dis. 2026 Mar 6;20(3):e0014029. doi: 10.1371/journal.pntd.0014029 (PMC12965539; doi:10.1371/journal.pntd.0014029)
Supplement: Attachment — Submitted filename: Response to Reviewers.docx [file pntd.0014029.s008.docx]

**Response to Reviewers**

Manuscript ID: PNTD-D-25-02000
Title: *From tests to truth: A misclassification-aware machine learning framework for estimating brucellosis seroprevalence in wild canids*

We would like to begin by sincerely apologizing for the delay in submitting our revised response. Due to the current situation in Iran, we experienced prolonged and severe internet disruptions, which posed substantial challenges during the revision process. Despite these difficult circumstances, we made every possible effort to address all comments with the highest level of care and accuracy, and to ensure that each response and revision was handled with full attention and precision.

We sincerely thank the Academic Editor, Section Editor, and all three reviewers for their thoughtful, constructive, and generous feedback. We are deeply grateful for the time and expertise they invested in improving our work. Below, we provide a detailed point-by-point response, and we have carefully revised the manuscript accordingly. If any additional clarifications or revisions are required, we would be grateful to be informed and will be pleased to address them.

**Response to the Editor**

**Editor Comment 1:**
“Your manuscript has been reviewed by 3 experts and each has provided suggestions and comments to improve your manuscript. Please evaluate their comments carefully, edit your manuscript to address those issues and resubmit with a ‘Responses to reviewers’ document.”

**Response:**
We thank the editor for coordinating the peer review process. We have carefully evaluated all reviewer comments, revised the manuscript accordingly, and provide a detailed, point-by-point “Response to Reviewers” document along with a tracked-changes version of the revised manuscript.

**Editor Comment 2:**
Please ensure that all CRediT author contributions are completed accurately and in full for all listed authors.

**Response:**
Thank you for the comment. We have carefully reviewed and completed the CRediT author contributions for all listed authors. The contributions have been updated to accurately and fully reflect each author’s role in the study, in accordance with the CRediT taxonomy.

**Editor Comment 3:**
Please provide an Author Summary (150–200 words) to appear between the Abstract and the Introduction.

**Response:**
An Author Summary of 173 words has been added to the manuscript and placed between the Abstract and the Introduction, in accordance with the PLOS Neglected Tropical Diseases guidelines.

**Editor Comment 4:**
Please upload all main figures as separate figure files in .tif or .eps format, following PLOS figure guidelines.

**Response:**
All main figures have been uploaded as separate figure files in **.**tif format, prepared in accordance with the PLOS figure guidelines.

**Editor Comment 5:**
Potential copyright issue identified for Figure 6B. Please provide:
(a) the source and direct link to the base map/shapefile, and
(b) license information confirming compatibility with CC BY 4.0.

**Response:**
Figure 7 has been recreated using R (renumbered from the original Figure 6B). The underlying basemap was obtained from **Natural Earth** (<https://www.naturalearthdata.com>), which provides public-domain geographic data fully compatible with the **Creative Commons Attribution 4.0 (CC BY 4.0)** license (<https://www.naturalearthdata.com/about/terms-of-use/>). The basemap source and license information have been added to the Figure 7 legend, and the Methods section has been revised accordingly. The full R script used to generate the figure is publicly available in the referenced GitHub repository.

**Editor Comment 6:**
Please update the Data Availability Statement to include a DOI/accession number or a direct link to each dataset, if available.

**Response:**
The Data Availability Statement has been updated to include a direct public link to the full analysis scripts and associated data, available at:
<https://github.com/ShamsFarzane/brucellosis-canids-seroprevalence.git>

**REVIEWER #1 — Point-by-Point Responses**

**1. Comment:**
Please rewrite your abstract into a non-structured abstract.

**Response:**
Thank you for this suggestion. The Abstract has been fully revised into a continuous, non-structured format, in accordance with the journal’s guidelines. The revised version presents the background, methodological approach, principal findings, and conclusions within a single coherent narrative, without section headings, while maintaining clarity and completeness.

**2. Comment:**
Please revise the following sentence as it appears to be AI-generated:
“While most control and surveillance programs have historically focused on livestock and human infections, increasing evidence indicates that wildlife—particularly free-ranging canids such as wolves (Canis lupus), foxes (Vulpes spp., Lycalopex spp.), jackals (Canis aureus), and coyotes (Canis latrans)—may play underappreciated roles in the ecology and transmission of *Brucella* species.”

**Response:**
Thank you for pointing this out. The sentence has been rewritten to improve clarity, tone, and readability and to better reflect conventional academic writing.

**3. Comment:**
Please delete all dots before citations and relocate them after the references throughout the entire manuscript.

**Response:**
Thank you for noting this formatting issue. All citation punctuation has been corrected throughout the manuscript to ensure consistent formatting.

**4. Comment:**
Line 95 contains an incorrect reference citation style.

**Response:**
Thank you for identifying this error. The citation format in this section has been corrected.

**5. Comment:**
Although you have an AI Disclosure, I suspect that many sentences are generated by AI. Please revise the entire manuscript.

**Response:**
We appreciate this concern. The AI Disclosure has been updated to clarify that AI tools were used only for grammar and language flow. In addition, the entire manuscript has been carefully reviewed and revised by the authors to improve clarity and readability and to reduce any AI-like phrasing. All substantive content, interpretation, and conclusions are solely the responsibility of the authors, and the revisions are provided with tracked changes.

**6. Comment:**
The introduction overstates the novelty of the modeling strategy without clearly distinguishing it from existing Bayesian disease-misclassification frameworks. Please clarify how your approach differs from established latent-class or hierarchical misclassification models in wildlife epidemiology.

**Response:**
Thank you for raising this important point. The Introduction has been revised to clarify how our framework relates to and builds upon existing latent-class and hierarchical misclassification approaches, and to more accurately contextualize its contribution.

**7. Comment:**
PCR and culture data are treated descriptively but not integrated into the modeling framework. Consider incorporating confirmatory data in a joint model or clearly justify why integration was not feasible.

**Response:**
Thank you for this important methodological comment. We have revised the Outcomes and unit of analysis section to explicitly clarify how confirmatory PCR and culture data were handled. We now state that active infection outcomes were analyzed on a separate diagnostic track and were used descriptively only, because confirmatory data were too limited to support integration into the Bayesian modeling framework. We further clarify that PCR and culture results were never combined with serological data, reflecting both biological differences between exposure and active infection and the fact that these diagnostics were applied to different individuals in nearly all studies.

**8. Comment:**
The text asserts that canids do not act as maintenance hosts, which may exceed the strength of the available data. Please rephrase conclusions to emphasize sentinel roles without excluding possible maintenance potential in specific contexts.

**Response:**
Thank you for this important comment. We have revised the Conclusion to avoid definitive statements regarding maintenance host status and to more accurately reflect the strength of the available evidence. The revised text now emphasizes that wild canids most consistently appear to act as sentinels of environmental and livestock-associated Brucella exposure, while explicitly acknowledging the possibility of localized maintenance in specific ecological or management contexts, particularly in captive or high-contact systems. This revision aligns the conclusions with the data and appropriately conveys uncertainty where evidence is limited.

**9. Comment:**
Several abbreviations (e.g., FPA, ICT, CIE) appear before being defined. Please ensure all diagnostic abbreviations are defined at first mention.

**Response:**
Thank you for pointing this out. We have revised the manuscript to ensure that all diagnostic abbreviations (including FPA, ICT, and CIE) are defined at their first occurrence in the text.

**10. Comment:**
The Results section includes interpretive statements that are better suited for the Discussion. Please restrict the Results to data presentation and move interpretive content to the Discussion.

**Response:**
Thank you for this helpful comment. We have carefully revised the Results section to ensure that it is restricted to descriptive reporting of data, estimates, and model outputs only. Interpretive statements, ecological explanations, and implications have been removed from the Results and consolidated in the Discussion section.

**REVIEWER #2 — Point-by-Point Responses**

**Comment 1:**
There are problems with the citations. The footnotes do not always follow the same convention with regard to punctuation. For example, on Page 5, three different footnote conventions are used:

- Line 90: “infection.[4]”
- Line 92: “settings. [5]”
- Line 98: “hosts[3,6].”
  Please review all footnotes to ensure they all use the same convention. Most are like the example from line 98.

**Response:**
Thank you for identifying these inconsistencies. We have reviewed all in-text citations throughout the manuscript and standardized citation punctuation to a single, consistent format, with citations placed immediately after the referenced text and punctuation applied uniformly. All spacing and punctuation inconsistencies have been corrected.

**Comment 2**

*There are several references that are incomplete.*

**Response:**
Thank you very much for this careful and constructive comment. We agree that accuracy and completeness of references are essential. We initially formatted the reference list using Zotero; however, following the reviewer’s observation, we re-examined the entire References section manually, one reference at a time, and revised all entries to ensure full completeness and strict compliance with PLOS Neglected Tropical Diseases (Vancouver/ICMJE) formatting requirements. All missing bibliographic details have now been added, and formatting inconsistencies have been corrected throughout the reference list.

**Comment 2a**

*Reference 30 is listed as: “Hoq MA. A serologic survey of Brucella agglutinins in wildlife and sheep. 1978.” No journal name, volume, issue, or page numbers are provided. An internet search suggests this may have been published in California Veterinarian, volume 32, pages 15–17.*

**Response:**
We thank the reviewer for identifying this omission. Reference 30 has been fully corrected by adding the complete journal information. It is now cited as:

**Hoq MA. A serologic survey of Brucella agglutinins in wildlife and sheep. *Calif Vet.* 1978;32(3):15–17.**

This reference now includes the journal name, volume, issue, and page numbers in accordance with PLOS standards.

**Comment 2b**

*Reference 35 is a doctoral thesis, and it is unclear whether the citation conforms to PLOS standards.*

**Response:**
We appreciate this clarification request. Reference 35 has been reviewed and confirmed to conform to PLOS guidelines for theses and dissertations, which require author name, title, degree type, institution, location, and year. The citation now reads:

**Tessaro SV. A descriptive and epizootiologic study of brucellosis and tuberculosis in bison in northern Canada. PhD dissertation. Saskatoon: University of Saskatchewan; 1987.**

This format is consistent with PLOS recommendations for non-journal scholarly works.

**Comment 2c**

*Reference 36 lists the author name in all capital letters, which is inconsistent with the formatting of other references.*

**Response:**
Thank you for noting this inconsistency. The author name in Reference 36 has been corrected to standard capitalization, and the reference now follows the same formatting as the rest of the list:

**Neiland KA. Further observations on rangiferine brucellosis in Alaskan carnivores. *J Wildl Dis.* 1975;11(1):45–53.**

**Comment 2d**

*Reference 43 is listed as: “Nymo IH, Fuglei E, Mørk T, Breines EM, Holmgren KE, Davidson RK, et al. Why are Svalbard Arctic foxes Brucella spp. seronegative? 2022.” No journal name, volume, issue, or page numbers are provided. An internet search suggests this may have been published in Polar Research, volume 41, article 7867.*

**Response:**
We thank the reviewer for this helpful suggestion. Reference 43 has been fully updated to include the complete publication details and now reads:

**Nymo IH, Fuglei E, Mørk T, Breines EM, Holmgren KE, Davidson RK, et al. Why are Svalbard Arctic foxes Brucella spp. seronegative? *Polar Res.* 2022;41:7867.**

This reference is now complete and compliant with PLOS citation standards.

**Comment 2e**

*References 47 and 48 also appear to be incomplete.*

**Response:**
We appreciate the reviewer’s attention to these references. Both References 47 and 48 have been carefully revised and completed according to PLOS guidelines for reports, including title, document type, publisher, location, and year. The corrected citations are:

**47. Pinigin AF, Zabrodin VA, Nikulina VI. Brucellosis in arctic foxes (Alopex lagopus). Report. Moscow: Academy of Sciences of the USSR; 1970.**
**48. Sachs R, Staak C, Groocock CM. Serological investigation of brucellosis in game animals in Tanzania. Report. Dar es Salaam: Veterinary Research Institute; 1968.**

**Comment 3**

*The reviewer did not exhaustively examine all references. The authors should examine the entire References section and ensure that all citations are complete and conform to PLOS standards.*

**Response:**
We fully agree with this recommendation and sincerely thank the reviewer for emphasizing this point. In response, we conducted a comprehensive manual audit of the entire References section (References 1–48). Although Zotero was used initially for reference management, each reference was subsequently checked and corrected manually, one by one, to ensure completeness, consistency, and full adherence to PLOS Neglected Tropical Diseases (Vancouver/ICMJE) reference formatting requirements. As a result, all references now include complete bibliographic information and consistent formatting.

**REVIEWER #3— Point-by-Point Responses**

We thank Reviewer #3 for their careful evaluation of our manuscript and for the detailed methodological and reporting recommendations. We address each comment point-by-point below.

**METHODS & REPORTING–RELATED COMMENTS**

**Comment 1:**
The authors state that they included studies that were “published between 1962 and 2025 in English, Persian, Spanish, Portuguese, or Russian.” However, the selection of the period 1962–2025 is not justified. In addition, in the limitations, the authors should acknowledge the language restrictions that could have limited the yield from the literature search.

**Response:**
Thank you for raising this point. We have clarified in the Inclusion and exclusion criteria section that no restrictions on publication year or language were applied during database searches, and that all retrieved records were screened regardless of date, language, or region of origin. The reported time span of 1962–2025 reflects the range of eligible studies identified through this unrestricted search rather than a predefined inclusion criterion. This clarification ensures that the reported period is understood as an outcome of the search rather than an imposed limitation.

**Comment 2:**
In accordance with PRISMA-ScR guidelines, the authors should provide the search terms and search string for at least one database. The current section on information sources lacks detail on how the records were searched, despite referring to Supplementary Material 2.

**Response:**
Thank you for this helpful suggestion. In accordance with PRISMA-ScR guidelines, we have revised the Methods section to explicitly report the complete search strategy for at least one database. The full PubMed search string is now included directly in the Bibliographic search strategy subsection as a representative example, detailing all controlled vocabulary terms and free-text keywords used. In addition, we clearly state that full database-specific search strings, filters, and syntax for all other sources are provided in Supplementary File S2. These revisions improve transparency and reproducibility of the literature search process.

**Comment 3:**
The authors state that “Inter-rater agreement was assessed during a calibration exercise on a random subset of studies and was high. Reasons for full-text exclusion were documented and summarized in the PRISMA diagram.” To enhance reporting transparency, the authors should include another supplementary file listing the reasons for exclusion of each of the 53 studies at the full-text review stage. It is also unclear how inter-rater agreement was assessed, and no results are presented in the Results section.

**Response:**
Thank you for this important comment regarding reporting transparency. We revised the Methods section to clarify that reasons for full-text exclusion are summarized in the PRISMA flow diagram (Figure 1) and are reported in detail in Supplementary Table S1. We also clarified that inter-rater agreement was assessed through a calibration exercise on a random subset of studies using Cohen’s kappa (κ), with disagreements resolved by consensus. In addition, we now report the quantitative result in the Results section: inter-rater agreement during the calibration exercise was high (κ = 0.86). These revisions improve transparency and fully document study selection decisions and reviewer agreement.

**Comment 4:**
In the data extraction section, the authors state that “Records sharing identical Study ID” were excluded. It is not clear what “identical” means. The authors should clarify which features were used to define identical records. If digital object identifiers (DOIs) were used, this should be explicitly stated.

**Response:**
Thank you for requesting clarification. We have revised the Methods section to clearly define what constitutes an identical record and the unit of analysis. A “population” is now explicitly defined as the unique combination of Study ID (first author and publication year), species, country, sampling year, and total sample size (N). Records were treated as identical only when all of these characteristics matched, indicating the same animals tested by multiple assays, in which case they were handled as multi-test populations. Records differing in any of these features were treated as distinct populations. DOIs were not used for record matching. These revisions clarify the criteria used for record identification, deduplication, and population definition.

**Comment 5:**
The description of how quality appraisal was conducted is limited and unclear. Specifically:

- How were the domains of the Newcastle–Ottawa Scale used to classify overall study quality as “Good,” “Fair,” or “Poor”?
- In Supplementary File 3, what do the codes “1” and “0” represent?
- In the final “QA” column, what do the numerical values represent, and what scale was used to interpret them?
- After quality appraisal, how was risk of bias assessed?
- How were the results of the risk-of-bias analysis integrated into the final interpretation of the results?
  The authors should provide a clear description in the Methods section. If possible, the authors should consider adding a traffic-light diagram as a supplementary file.

**Response:**
We thank the reviewer for this important comment. We have substantially expanded and clarified the **Quality appraisal** subsection in the Methods to fully describe the adapted Newcastle–Ottawa Scale, including the three assessment domains, the seven binary items, and the scoring scheme. We now explicitly state that each item was scored as 1 (criterion met) or 0 (criterion not met), that total scores ranged from 0 to 7, and that these summed scores are reported in the final “QA” column, with higher values indicating lower risk of bias. Clear thresholds are provided to classify studies as low, moderate, or high risk of bias.

In addition, we added a dedicated **Risk of Bias Assessment** subsection in the Results to describe how quality appraisal findings were synthesized, including the number of studies in each risk category, common sources of bias, geographic patterns, and associations with diagnostic methods. We also clarify how risk-of-bias patterns were used to contextualize regional and diagnostic-specific findings and were examined in sensitivity analyses.

Full item-level scoring is provided in Supplementary Table S4, and we added a graphical summary of risk-of-bias domains as **Supplementary Figure S5**, addressing the suggestion for a traffic-light–style visualization. Together, these revisions provide a transparent and comprehensive description of quality appraisal and its role in interpretation.

**RESULTS REPORTING**

**Comment 6:**
Before synthesizing the results, the authors should include a “Search Results” section showing the number of sources screened, assessed for eligibility, and included in the review, with reasons for exclusion at each stage. This section should refer to the supplementary material listing reasons for exclusion of each of the 53 excluded studies. The PRISMA diagram should be cited and included with a clear caption. In the text preceding the PRISMA diagram, all included studies should be cited.

**Response:**
Thank you for this recommendation to strengthen PRISMA reporting. We revised the Methods section to explicitly link the PRISMA flow diagram to the screening process and full-text exclusion documentation. The revised text now states that the PRISMA diagram summarizes record identification, screening, and full-text assessment (Figure 1), while detailed reasons for exclusion are provided in Supplementary Table S1. This ensures internal consistency between the Methods narrative and the accompanying PRISMA and supplementary materials.

**Comment 7:**
The authors should include a section describing the characteristics of the studies included. This section should refer to Table 1, which needs improvement. It is unclear what the column labeled “Total” represents and this should be clarified. A column for sample size should be included. If the “QA” column refers to quality appraisal, the meaning of those values should be explained in the table footnote.

**Response:**
Thank you for this helpful suggestion. We have added a dedicated **“Characteristics of Included Studies”** section in the Results that summarizes the number of studies and populations, geographic coverage, host species, and diagnostic methods, with explicit reference to **Table 1.**

In addition, **Table 1 has been revised** to improve clarity and transparency. The column previously labeled “Total” has been renamed **“N (sample size)”** and is explicitly defined as the total number of animals tested in each study arm. The meaning of the **“QA”** column is now clearly explained in the table footnote, including the scoring range, risk-of-bias categories, and interpretation of values. These revisions address the reviewer’s concerns and improve the readability and interpretability of the table.

Because quality appraisal criteria were clarified during revision, the quality assessment was reapplied consistently across studies, and the updated QA scores are now reported uniformly in the table and results text. These revisions address the reviewer’s concerns and improve the readability, transparency, and interpretability of the study characteristics and quality assessment.

**Comment 8:**
The authors should include a Risk of Bias section providing a synthesis of the findings from the risk-of-bias analysis. This section should clearly indicate which studies have low, moderate, or high risk of bias, and the results of the risk-of-bias analysis should be integrated into the results discussion.

**Response:**
Thank you for this important suggestion. We have addressed this comment in two complementary ways. First, we added a dedicated **Risk of Bias Assessment** subsection within the Results, which summarizes the findings of the risk-of-bias evaluation across all included studies and clearly reports the number of studies classified as low (n = 21), moderate (n = 12), and high risk (n = 4) based on predefined Newcastle–Ottawa Scale thresholds. This section also synthesizes key patterns by region and diagnostic approach.

Second, the results of the risk-of-bias analysis have been explicitly integrated into the **Discussion**, where we interpret regional and diagnostic heterogeneity in light of methodological limitations identified through the appraisal. In particular, we highlight the influence of legacy serological assays and under-sampled regions on observed prevalence patterns. Together, these revisions ensure transparent reporting of risk-of-bias findings and their appropriate incorporation into the interpretation of results, as requested.

**CONCLUSIONS**

**Comment 9:**
The conclusions should be revised based on the results of the risk-of-bias analysis.

**Response:**
Thank you for this valuable recommendation. The Conclusion has been revised to explicitly incorporate the findings of the risk-of-bias assessment. We now emphasize that observed variation in Brucella exposure among regions and species is strongly influenced by methodological factors, including diagnostic assay choice and uneven geographic coverage, particularly the reliance on older serological tests and limited data from Africa and parts of Asia. The revised text avoids overinterpretation of apparent patterns and frames the conclusions in light of identified biases and data gaps, thereby aligning inference with the quality and limitations of the underlying evidence.

**Comment 10:**
In the abstract (lines 50–51), the authors should clarify which exposure is being referred to (human or wild canids). In the methods portion of the abstract, the authors should describe how records were obtained and from which databases. The statement “Across 48 wild populations (N = 3,925 animals)” should clarify which populations are being referred to.

**Response:**
We thank the reviewer for this important comment. The Abstract has been revised to improve clarity and specificity throughout. We now explicitly state that the analysis focuses exclusively on free-ranging wild canids, with no inclusion of human data. The study identification process has been clarified by explicitly naming the databases searched (PubMed, Scopus, Web of Science, SciELO, and Google Scholar) and the study period. In addition, we have clarified that the phrase “48 wild populations” refers to distinct wild canid serological populations included in the synthesis, with the total sample size clearly specified. These revisions ensure that the exposure definition, data sources, and study populations are unambiguous to the reader.

**Closing Statement**

We gratefully thank the Academic Editor, Section Editor, and reviewers for their thoughtful, constructive, and detailed feedback. Their insightful comments and suggestions have substantially strengthened the clarity, rigor, and overall quality of the manuscript. We sincerely hope that the revised version now meets the expectations of PLOS Neglected Tropical Diseases. We remain fully available to provide any further clarifications, revisions, or additional information should they be required, and we greatly appreciate the opportunity to improve our work through this rigorous review process.

With sincere appreciation,

**The Authors**
